# Global Insights Into Rural Health Workers' Job Satisfaction: A Scientometric Perspective

**DOI:** 10.3389/fpubh.2022.895659

**Published:** 2022-06-17

**Authors:** Yuquan Chen, Yanwei You, Yiche Wang, Yutong Wang, Tao Dai

**Affiliations:** ^1^Institute of Medical Information/Library, Chinese Academy of Medical Sciences, Beijing, China; ^2^Peking Union Medical College, Beijing, China; ^3^School of Social Sciences, Tsinghua University, Beijing, China; ^4^Department of Economics and Management, Shanghai University of Sport, Shanghai, China; ^5^School of Health Preservation and Rehabilitation, Chengdu University of Traditional Chinese Medicine, Chengdu, China

**Keywords:** rural health workers, job satisfaction, scientometric, web of science, job burnout

## Abstract

**Introduction:**

Rural health workers (RHWs) play an irreplaceable role in ensuring and improving the health level of rural residents as the most basic and extensive medical service providers in rural areas. However, rural health institutions are facing significant worker shortages worldwide, not only in low- and middle-income countries but also in developed countries. As an important variable to explain RHWs' work status and predict turnover behavior, job satisfaction has received more and more attention currently.

**Methods:**

Publications from 1 January 1995 to 31 December 2021 were identified from the Science Citation Index Expanded (SCI-Expanded), the Social Sciences Citation Index (SSCI), and the Emerging Sources Citation Index (ESCI) of the Web of Science Core Collection (WoSCC); CiteSpace, VOSviewer, and R software were applied to conduct this study.

**Results:**

A total of 251 publications were obtained from the WoSCC database. The number of publications had a statistically significant increase in the study period. Ranking in the top three of the most productive countries or regions in this field was the United States, the United Kingdom, and China. “Health Care Sciences & Services,” “Nursing,” and “Public, Environmental & Occupational Health” seemed to be the major subjects. According to the reference co-citation analysis, “motivation,” “rural and remote areas,” and “work environment” were three noteworthy topics during the development of the research field. Moreover, through the keyword analysis, the underlying relationship among “job satisfaction,” “job burnout,” and “turnover intention” was explored.

**Conclusion:**

Publications about job satisfaction associated with RHWs had remarkably indicated that this research field had great development potential and broad prospects. As an emerging topic related to RHWs' job status, job satisfaction and its related affected factors were systematically summarized by cluster and keywords analysis. We also highlighted that job satisfaction had a negative predictive effect on RHWs' job burnout and turnover intention, and job burnout played a positive role in predicting turnover intention. In addition, the job satisfaction and working environment of RHWs under the COVID-19 pandemic should receive more attention in the future.

## Introduction

Rural health workers (RHWs), including doctors, nurses, public healthcare workers, and administrative staff, play an irreplaceable role in ensuring and improving the health level of rural residents as the most basic and extensive medical service providers in rural areas. They offer a series of primary healthcare services, including the establishment of rural health archives, education of health knowledge, prevention and control of infectious diseases, healthcare for the elderly, and management of chronic diseases (1–3). However, rural health institutions are facing significant worker shortages worldwide, not only in low- and middle-income countries (4, 5) but also in developed countries (6, 7).

Job satisfaction is the extent to which people like (satisfaction) or dislike (dissatisfaction) their jobs and refers to an attitude or emotional response to one's tasks as well as to the physical and social conditions of the workplace (8–10). It is one of the most important predictors of medical staff burnout and refers to a series of psychological and physiological reactions caused by the pressure of the interpersonal relationship and work itself (11). Turnover intention refers to the idea that an individual has to resign from his current job and look for another job (12). Job burnout, which is a kind of psychological syndrome (11), refers to a series of psychological and physiological reactions caused by the pressure of the interpersonal relationship and work itself. Some studies have explored the relationship among them, e.g., a survey of 1,148 rural primary care providers (PCPs) found that there was a significant direct effect of job satisfaction on burnout and turnover intention and a significant indirect effect of job satisfaction on turnover intention through burnout as a mediator (13).

The current situation of RHWs' job satisfaction around the world has its own characteristics. Among rural American physicians, the increased workload and longer hours contributed to lower job satisfaction, poor retention rates, and decreased physician wellness (6). Another national survey indicated that the proportion of the rural nursing workforce in Canada is continuing to decline in relation to the proportion of the Canadian population in rural and remote settings, although their levels of satisfaction with their nursing practice and community are generally high (7). Job satisfaction among RHWs in developing countries cannot be ignored, e.g., China, which had a rural resident population of 509.79 million by the end of 2020; a job satisfaction survey of 5,046 RHWs in 11 provinces found that the average score of the overall job satisfaction of RHWs was 3.20 ± 0.55 (total score was 5.00), namely with a degree of 64.1%, indicating that the RHWs were slightly satisfied with their jobs, and job satisfaction proved to be negative predictors of turnover intention (14). Similarly, in rural British Columbia, the job satisfaction of physicians was also low, and a 7-point Likert-type scale showed that its average score was only 3.7 ± 1.0 combined with a 55% self-reported burnout rate.

In recent years, the high turnover rate of RHWs all over the world, especially in developing countries, has hit the healthcare system in rural areas, and the decline in job satisfaction makes them lack enthusiasm and willingness to strive to provide high-level services, which indirectly hinders health promotion and increases their tendency to leave (15, 16). However, compared with their colleagues who are in urban secondary or tertiary public hospitals, they might benefit less from their current job (17, 18), and it seems that there are more relevant studies (19). Nonetheless, for RHWs who are equally important, the current situation of important factors affecting turnover behavior and work efficiency, namely, job satisfaction, is not only the inadequate original survey but also insufficient medical staff (20). In this condition, it is urgent to explore the risk factors affecting RHWs' poor job satisfaction and find their internal relationship. Unfortunately, few scholars have comprehensively evaluated the current situation and detected the underlying reasons from a global insight. Therefore, our team plans to undertake this important topic to make up for the gaps and defects in this research field.

## Materials and Methods

### Data Source and Search Strategy

**Figure 1:**
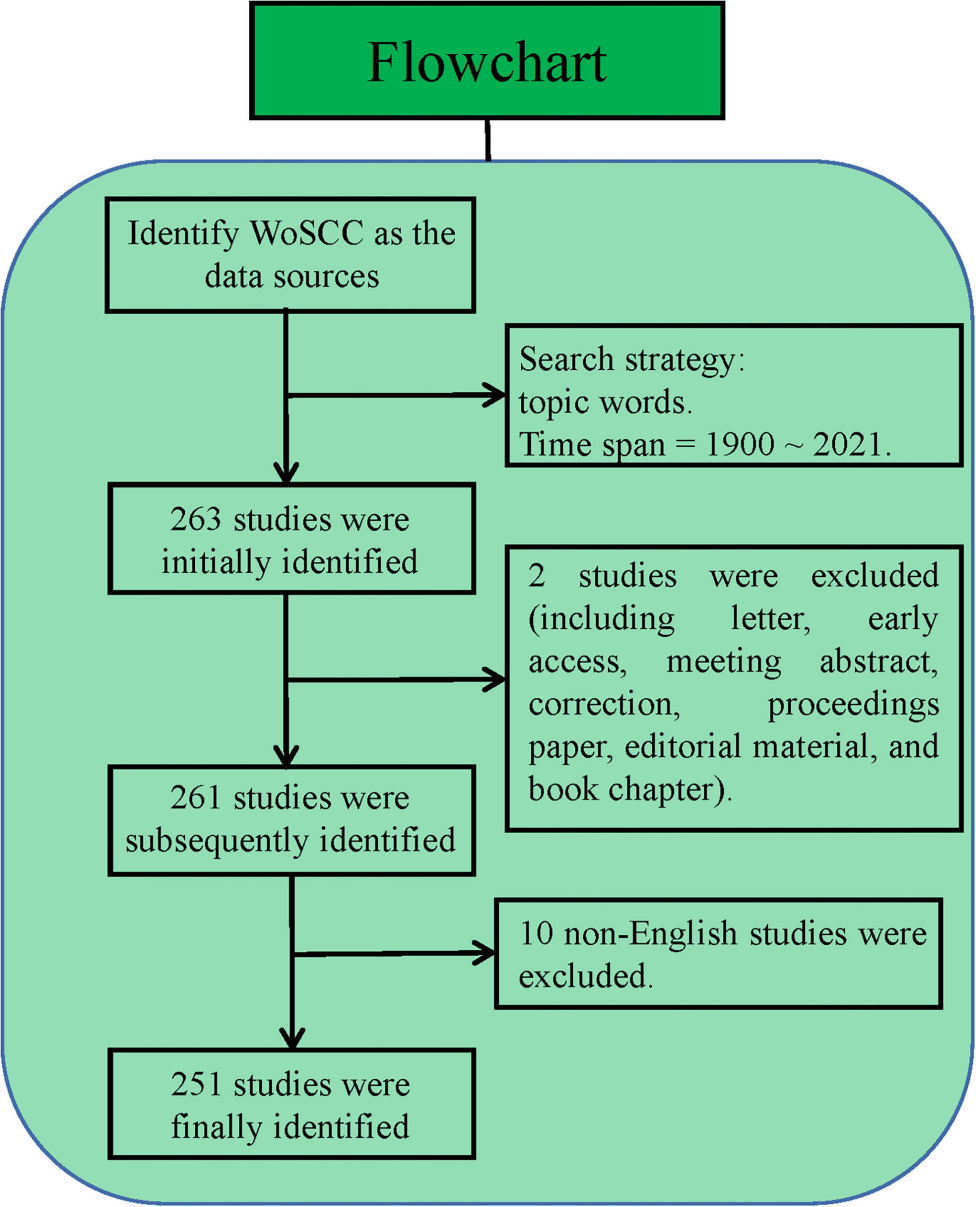
Summary of retrieval framework.

All publications were retrieved online through the Science Citation Index Expanded (SCI-Expanded), the Social Sciences Citation Index (SSCI), and the Emerging Sources Citation Index (ESCI) of the Web of Science Core Collection (WoSCC) on 3 January 2022. The search strategy was based on a combination of [TS=(rural OR countryside OR district OR community OR village OR grassroots)] AND [TS=(doctor OR physician OR nurse OR practitioner OR “health worker” OR “health officer” OR “health personnel” OR “medical personnel” OR “medical worker” OR “medical staff” OR “physician assistant”)] AND [TS=(“job satisfaction” OR “work satisfaction” OR “professional satisfaction” OR “career satisfaction” OR “job burnout” OR “turnover intention” OR “departure intention” OR “demission intention” OR “job demission intension” OR “turnover tendency” OR “departure intention tendency” OR “demission tendency” OR “job demission tendency”)] AND [TS=(“risk factor” OR influence OR correlate^*^)]. The two authors, YC and YY, participated in the material search and data extraction process. The pre-retrieval results found that there was no publication before 1 January 1995; hence, the search time frame in this study was set from 1 January 1995 solstice to 31 December 2021. According to previous literature (21–23), “articles or reviews” were used as inclusion criteria, and the language was limited to “English.” Related data were extracted and downloaded within 1 day to avoid bias due to frequent database updates. Basic information of each research included was retrieved as “full record and cited references” for further analysis. The retrieval framework is shown in Figure 1.

### Analysis Tools

The research methods used in this article were scientometric and bibliometric strategies. Scientometric is an emerging way that can both quantitatively and qualitatively analyze the research trends and study the status of certain topics by using mathematics and statistics methods. Compared with traditional meta-based analysis, scientometrics or bibliometrics have a relatively broad horizon of the current hotspots and research domains (22). In this study, two Java-based visualization tools, namely, CiteSpace and VOSviewer, were applied to reveal the results from bibliometric analysis. Visualization of knowledge domains, collaborative networks among regions and institutions, research categories, and reference co-citations were analyzed, and then the landmark literature, influence factors, and implicated enlightenment of the job satisfaction of RHWs were presented.

Referring to the classic settings by Chaomei Chen, the designer of CiteSpace, the basic parameter of CiteSpace was set as follows: link retaining factor (LRF) = 1, maximal link per node (L/R) = 10, look back years = 5, and top *N* [*n* | *f* (*n*) ≥ *e*] = 1, the scale factor *k* = 25. In addition, the network and overlay visualization was also set by the VOSviewer manuals. The co-occurrence function of VOSviewer was applied to explore the keyword associations, the counting method was set as full counting, and the unit of analysis was author keywords (AKs). The size variation determined the strength of the connection effect by each node. Association strength was used for normalizing the strength of the links between items, and this method was identical to the Van Eck and Walkman's criteria (24).

In the collaboration analysis, each node in a map represents an individual and the size of the node speaks on behalf of the centrality (25, 26). A larger size indicates higher occurrence or citation frequency. There are a number of measurements to calculate the centrality index, namely, eigenvector centrality, closeness centrality, and degree. Referring to previous literature, the centrality applied in this study was the classic betweenness centrality (27). When it comes to the lines, each line builds a bridge between two nodes and reflects connections of them. Similarly, the wider the line, the stronger the connection between two nodes.

The *R* software (version 4.0.3) was used for the co-occurrence keywords analysis. The cosine similarity algorithm was applied to generate the keywords' co-occurrence matrix. A heat map was provided to reflect the degree of inter-relationship of the high occurrence keywords. The Microsoft Excel 2016 was used to describe the research trend. The function model was set as follows: *f(x)* = *ax*^3^ + *bx*^2^ + *cx* + *d*. In this function, *x* represented the publication year and *f* (*x*) showed the cumulative amount. In this way, we conducted a scientometric analysis on job satisfaction of RHWs' research in the past several decades to discover the research status, current hotspots, and influence factors, which can further provide researchers and healthcare policy makers with meaningful advices.

## Results

### Annual Outputs Analysis

**Figure 2:**
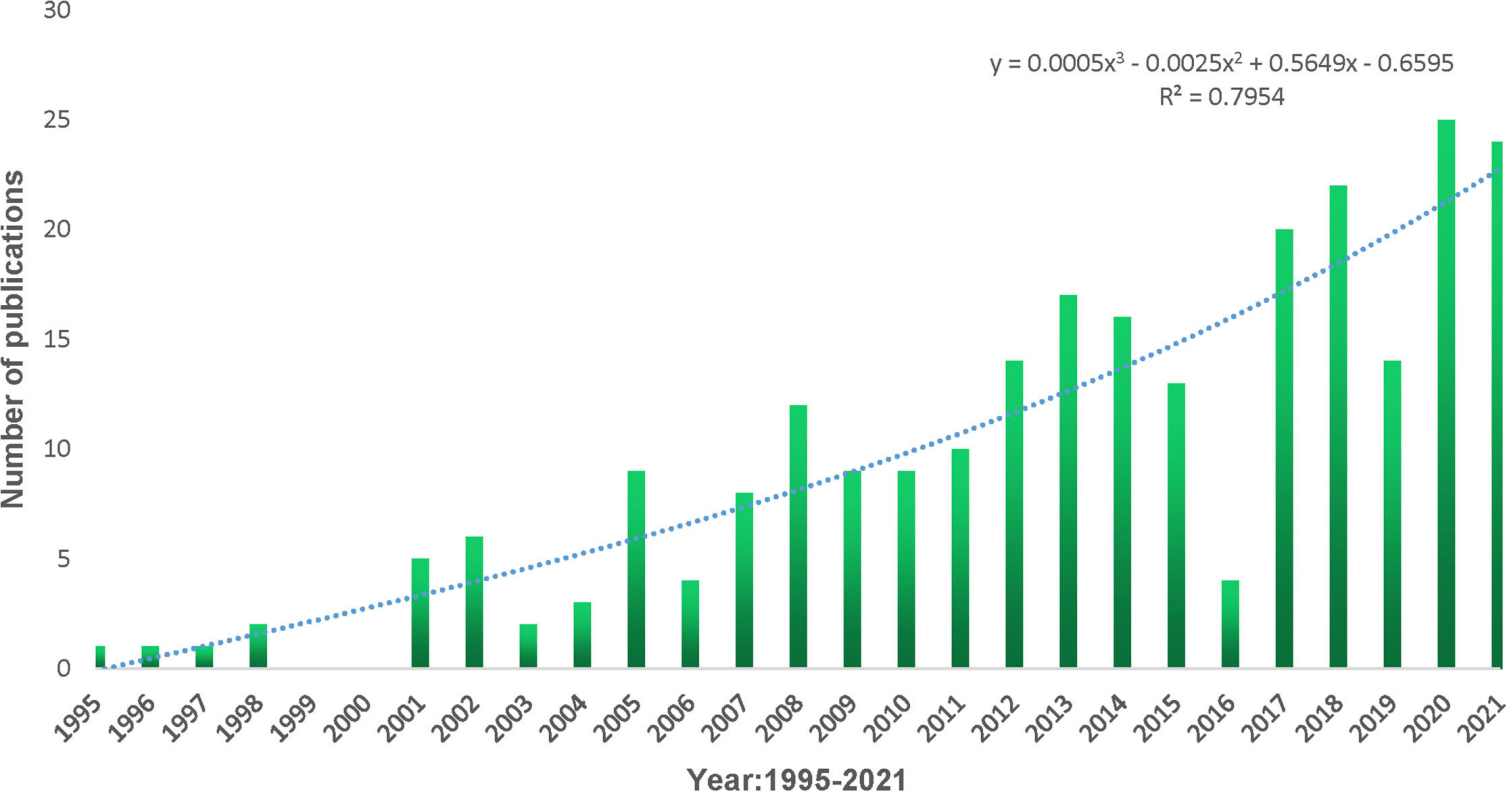
Annual output of publications and growth trends.

A total of 251 publications between 1995 and 2021 met the inclusion criteria. Two studies (one early assessment and one editor material) and 10 non-English papers were excluded. Figure 2 represents the annual publishing trend. On the basis of the statistical analysis, results showed that the number of literatures rose from 1 in 1995 to 24 in 2021. We performed the linear regression analysis and confirmed that the percentage of publications had a statistically significant increase in the study period (*P* < 0.001, *t* = 8.374). However, it should be noted that the number of publications in 2016 and 2019 decreased compared with adjacent years.

### Analysis of Countries and Institutions

**Figure 3:**
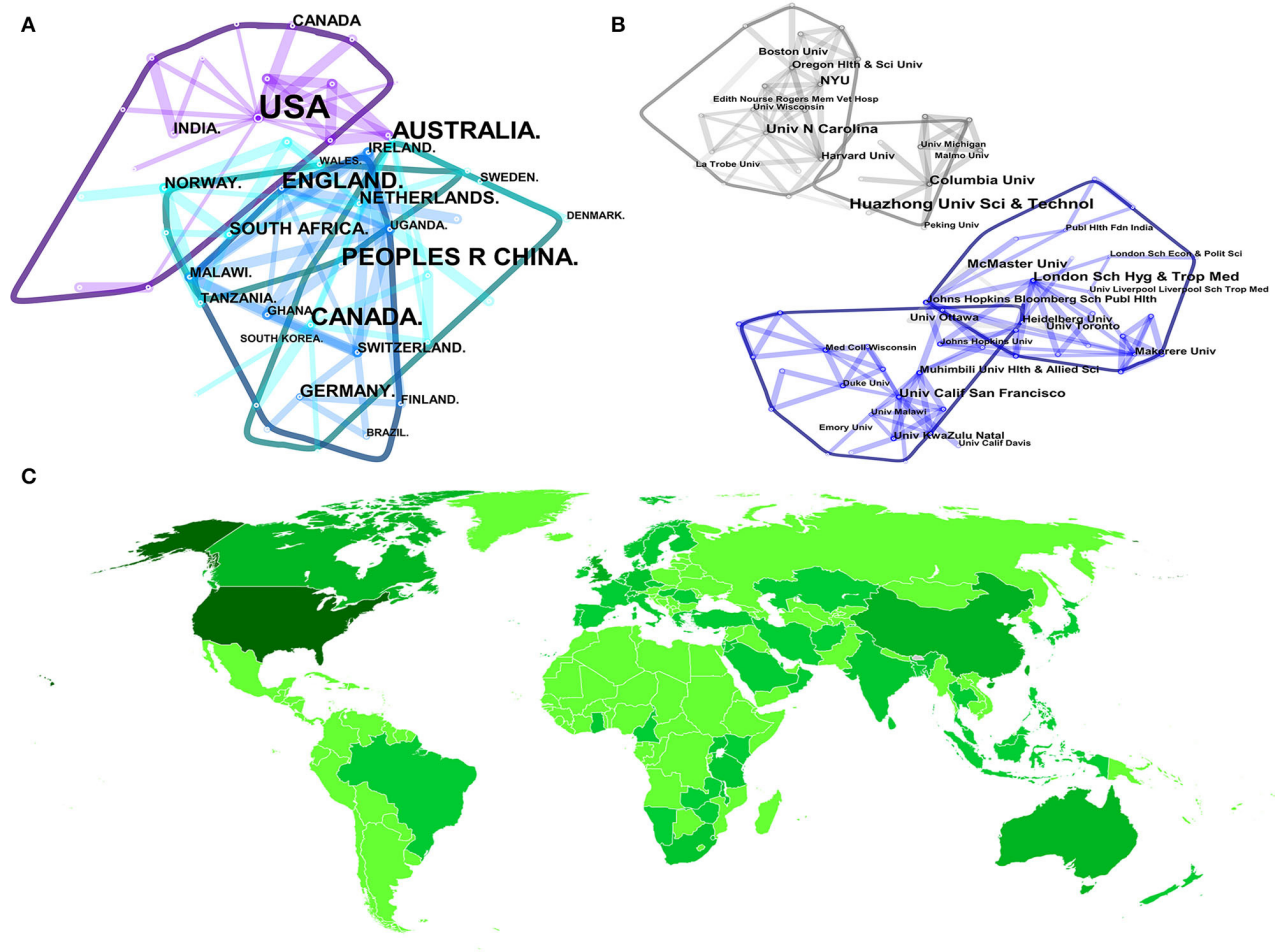
Map of countries **(A)**, institutions **(B)**, and world distribution **(C)** of publications in the research field.

All studies included in this study were produced by 70 different countries. The collaboration status was generated by CiteSpace with 70 nodes and 113 links, which indicated that the total publications were published in 70 countries or regions with 113 connections among them. Figure 3A reflects the five most prolific countries related to job satisfaction of RHW groups, with the top three from the United States (86, 34.2%), the United Kingdom (26, 10.4%), and China (24, 9.6%). From Table 1, it can be found that the top five countries' contributions have made up over 70% of total publications, which indicated that they contributed chiefly to the research domain.

**Table 1:** Ranking of top five countries and institutions of the research domain.

**Rank**	**Country**	**Publications**	**Percentage**	**Rank**	**Institution**	**Publications**	**Percentage**
1	United States	86	34.2	1	Johns Hopkins Univ	7	2.8
2	United Kingdom	26	10.4	2	Huazhong Univ Sci & Technol	6	2.4
3	China	24	9.6	3	Columbia Univ	5	2.0
4	Australia	22	8.8	4^**^	London Sch Hyg & Trop Med	5	2.0
5^*^	Canada	22	8.8	5^**^	Harvard Univ	5	2.0

* *Indicates a tie for fourth place*.

** *Indicates a tie for third place*.

A total of 380 different institutions contributed to RHWs' career satisfaction during the past four decades. Figure 3B details the relationship among these institutions with 380 nodes and 445 links. Johns Hopkins University engaged in the most studies with seven documents, followed by Huazhong University Science & Technology with six literatures. Besides that, Columbia University, London School of Hygiene & Tropical Medicine, and Harvard University were also productive institutions with the same six publications, respectively. Among these five institutions, three of them were in the United States, and one of which was located in the United Kingdom and China, respectively. However, different partners seemed to have weak academic collaborations with each other, which might partially be due to the total amount of the research and insignificant attention.

Figure 3C represents the degree of contribution among the regions engaged in this domain from a global scale. From Figure 3C, it can be seen that countries and institutions in North America, Western Europe, and East Asia were dominated in the RHWs' job satisfaction field.

### Analysis of Categories and Journals

**Figure 4:**
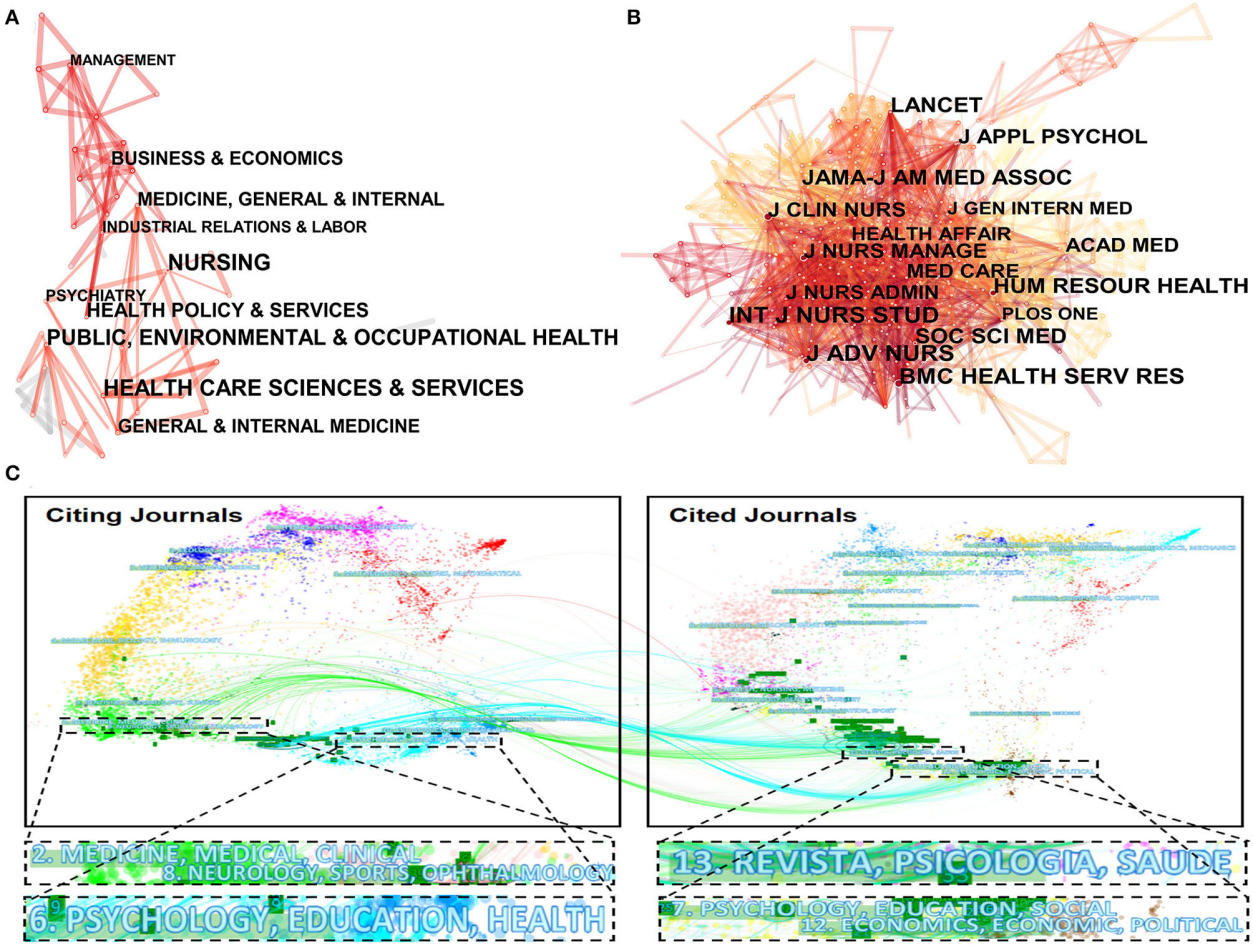
Map of disciplines **(A)**, cited journals **(B)**, and dual-map overlay **(C)** of publications in the research field.

The subject category could help researchers better know the focus and trends of recent studies (28). Subjects involved in publishing literature on RHWs' job satisfaction are displayed in Figure 4A. According to Figure 4A, it was obvious that “Health Care Sciences & Services,” “Nursing,” and “Public, Environmental & Occupational Health” seemed to be the major subjects. The subject network was made up of 63 nodes and 118 links, which indicated that 63 sub-disciplines participated in this research field with 118 connections built closely.

Subsequently, journals in the research field were analyzed. In this research, we defined journals as citing journals and cited journals. Citing journal was the journal publishing papers in the field of RHWs' job satisfaction, and cited journal was the journal where the references cited in this research field were published. The top 10 active journals published 85 papers, which accounted for 33.86% of the total publication outputs (Table 2). Figure 4B demonstrates the status of cited journals. In Figure 4B, 471 nodes and 2,939 links were built simultaneously, which meant that there were 471 cited journals in this domain. From Table 2, we can find that *Journal of Advanced Nursing* led the way with 82 co-citation counts, and *International Journal of Nursing Studies* and *BMC Health Services Research* also contributed to 78 and 73 counts, respectively. A famous international medical journal, including *LANCET* and *JAMA* sub-journal, also contributed to the top 10 co-citation counts. All these top 10 citing and cited journals were recognized as mainstream journals in the domain of RHWs' job satisfaction.

**Table 2:** Ranking of top 10 journals and co-cited journals.

**Rank**	**Citing journal**	**Publications**	**Rank**	**Cited journal**	**Co-citation counts**
1	Human Resources for Health	16	1	Journal of Advanced Nursing	82
2	BMC Health Services Research	15	2	International Journal of Nursing Studies	78
3	Journal of Nursing Management	13	3	BMC Health Services Research	73
4	Rural and Remote Health	7	4	Human Resources for Health	62
5	Journal of Nursing Administration	6	5	Social Science & Medicine	61
6	Journal of Rural Health	6	6	LANCET	57
7	BMC Family Practice	5	7	JAMA-Journal of the American Medical Association	57
8	International Journal of Nursing Studies	5	8	Journal of Applied Psychology	53
9	Academic Medicine	4	9	Journal of Clinical Nursing	51
10^*^	BMJ Open	4	10	Journal of Nursing Management	49
11^*^	International Journal of Environmental Research and Public Health	4			

* *Indicates a tie for ninth place*.

Figure 4C shows a dual-map overlay of the relationship between citing journals and cited journals in the research domain. In Figure 4C, there were two major citation paths worthy of attention. The green paths represented that research published in “medicine, medical, clinical” and “neurology, sports, ophthalmology” journals preferred to quote journals in the domains of “Revista, Psicolologia, Saude (Portuguese).” The blue paths showcased that studies published in “Psychology, Education, Health” journals tended to cite journals mostly in the domains of “Psychology, Education, Social” and “Economics, Economic, Political.” It is noteworthy that RHWs' job satisfaction is actually a comprehensive field, and more interdisciplinary collaborations are pending to promote.

### Reference Co-Citation Analysis

**Figure 5:**
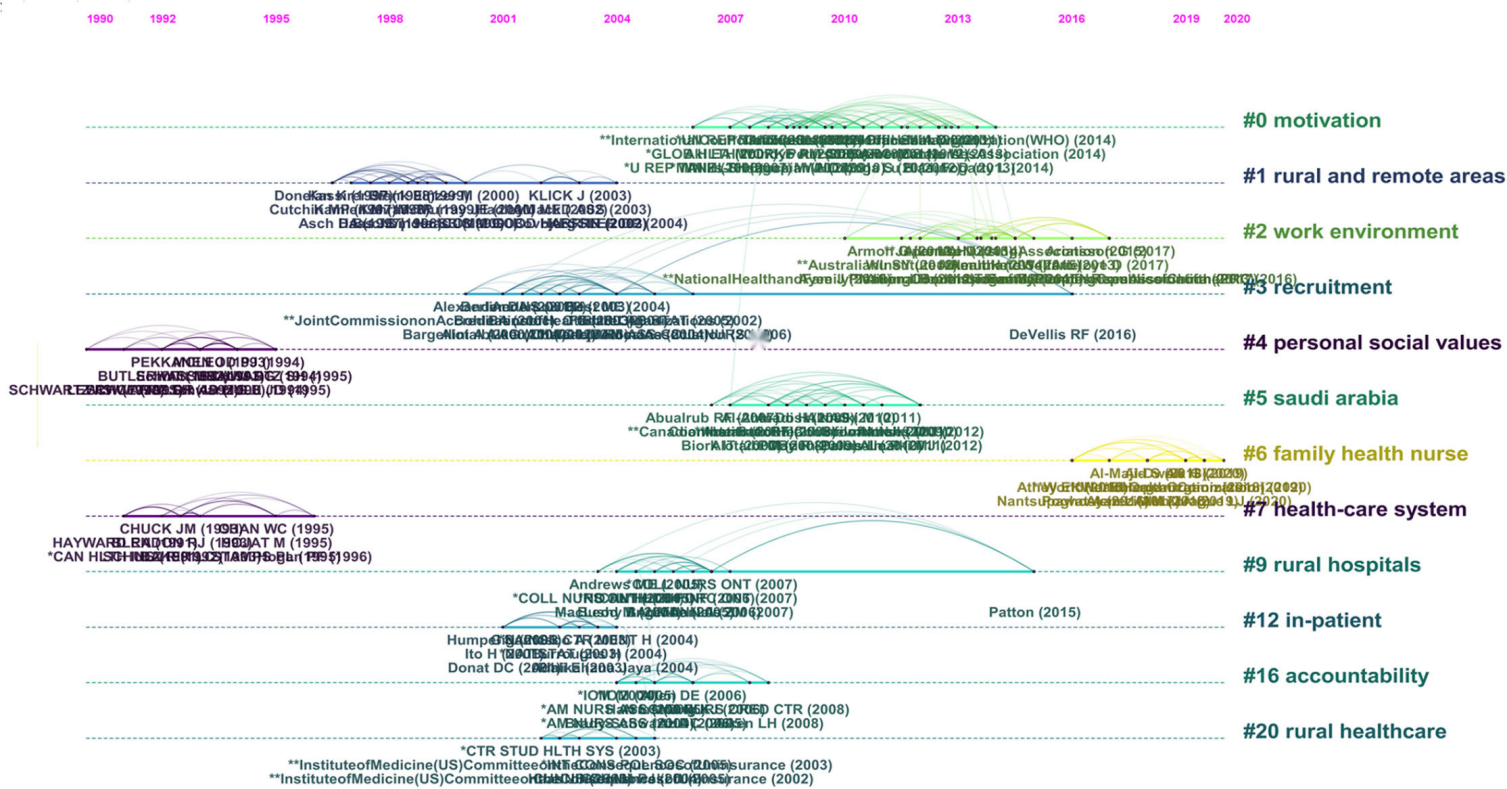
The timeline view of the knowledge map is based on reference co-citation analysis.

The analysis of co-cited references can help us know the citation status of different scientific documents (26). The co-citation index was thought to be a critical indicator in several previous knowledge map studies that allows the researcher to better understand hotspots in specific research fields. Among algorithms to generate reference co-citation clusters, the log-likelihood ratio was applied in this study that can cover the “uniqueness and coverage” of all labels created. Figure 5 represents the top 12 clusters in a timeline view. Modularity value (*Q* value) and weighted mean silhouette value (*S-*value) were applied to assess the rationality of clustering. Referring to the *Q* and *S* reported in the previous literature (29), the *Q* in our study equaled 0.9566 and *S* equaled 0.9889, which represented a relatively high level of clustering homogeneity, which in other words verified the rationality of clustering. In Figure 5, all clusters were labeled by index terms extracted from the references. The top three clusters were “motivation” #0, “rural and remote areas” #1, and “work environment” #2, respectively.

### Keywords Analysis

**Figure 6:**
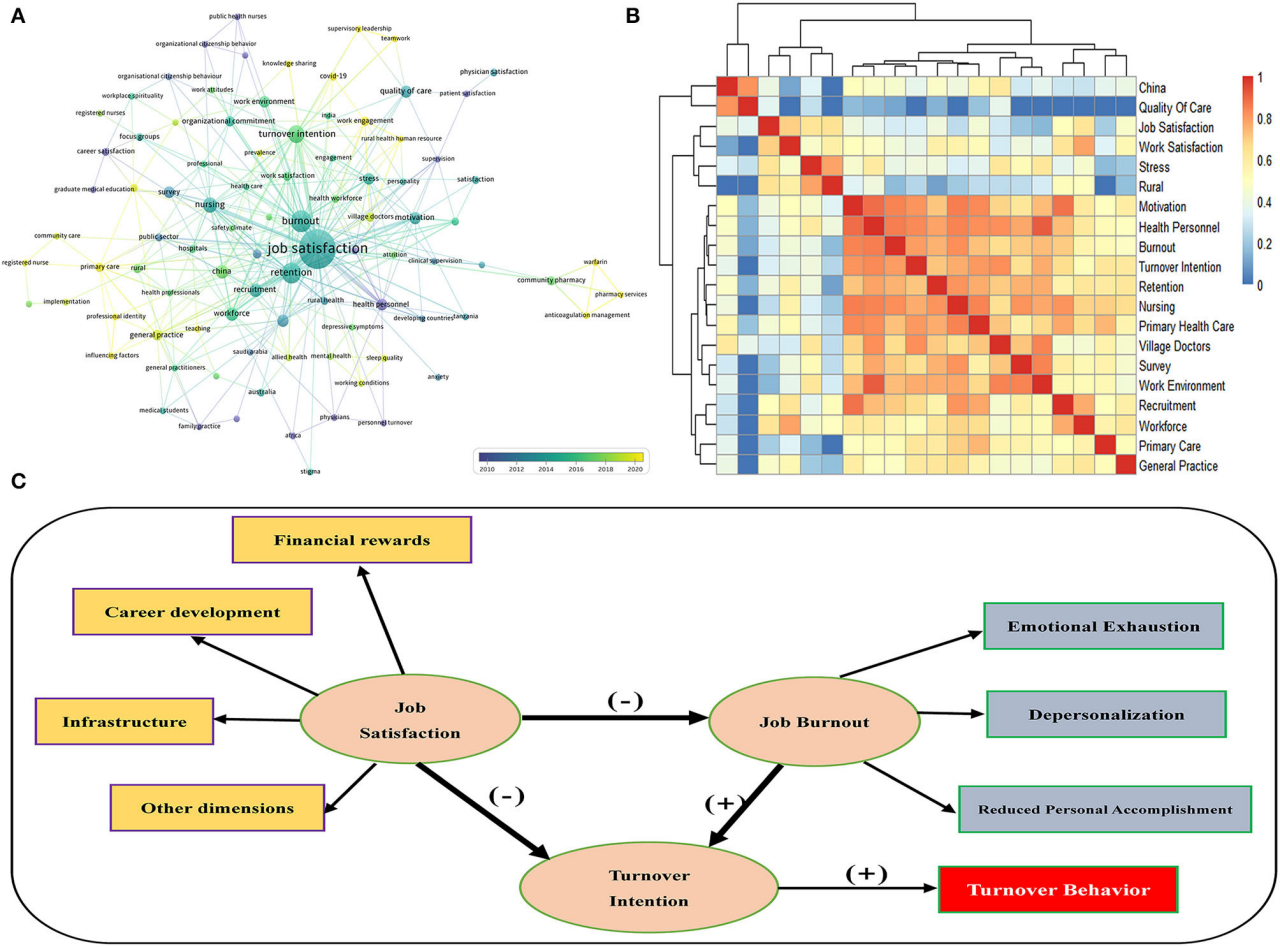
The co-occurrence map **(A)**, heat map **(B)**, and relationship **(C)** of high-frequency keywords.

Keywords can reflect major interests and current tendencies of a knowledge field. The VOSviewer software was applied to generate a network map of the co-occurrence keyword. By using a thesaurus to clean and purify the data, 92 keywords met the threshold and are presented in Figure 6A. There were 321 connections among these keywords, and the thickness of links demonstrated the occurrence frequency. Among all keywords included, “job satisfaction” (121 times), “retention” (62 times), “burnout” (42 times), and “turnover intention” (37 times) were the top 4 high-frequency keywords.

We next investigated the correlation among the top 20 keywords. The *Pheatmap* package of the *R* software 3.6.3 was used to generate the heat map. As shown in Figure 6B, *k*-means clustering algorithm was further applied to cluster these keywords included. In this figure, we can divide all clusters into three parts, the first part included “China” and “Quality of care”; the second part included “Job satisfaction,” “Work satisfaction,” “Stress,” and “Rural”; and the third cluster included the remaining 14 keywords. Moreover, the first cluster reflected the area and districts in this research field and the second part emphasized the most concerned central phrases. Meanwhile, the third cluster mainly identified and illustrated the influencing factors of this research topic. We could find that people especially in China represent RHWs, such as rural health personnel and village doctors, whose job or work satisfaction was more relevant to their turnover intention, burnout, workforce, work environment, and so on. In addition, the quality of care especially primary healthcare seemed to have a stronger correlation with RHWs' job satisfaction and its corresponding influencing factors.

From Figures 6A,B, we further concluded the underlying relationship among these keywords. As shown in Figure 6C, the relationship can be simplified into three subjects, namely, “Job satisfaction,” “Job burnout,” and “Turnover intention.” We could predict research frontiers through an in-depth understanding of the relationship among these keywords. The detailed analysis of relationship and influencing factors of these keywords are discussed in the next section.

## Discussions

This study provides a bibliometric analysis of publications on global RHWs' job satisfaction from 1995 to 2021. Based on the annual output analysis, we could find that the publication–year distribution has experienced a rapid growth during the past 27 years. Thus, studies on this issue have received increasing attention. Obviously, the developed countries represented by the United States and Britain have undertaken most of the research on this issue. As a country with 509.79 million rural people, accounting for 36.11% of the total population (30), Chinese studies on this important issue has only ranked third, even though this number has ranked first in developing countries. Simultaneously, more and more authoritative journals with high impact factors have participated in the discussion on RHWs' job satisfaction and, hence, this issue has also become more important.

From regional analysis, we can further get information about the global status of this special topic. Although RHWs were common in developing countries, institutions in developed regions seemed to invest more resources in caring about the job satisfaction of this special population. Strikingly, as the largest developing country, China also made specific contributions in this domain. China faces arduous challenges in the rapid loss of RHWs. A recent meta-analysis indicated that the prevalence of turnover intention of rural primary healthcare workers was about 23.4%−42.1% (28). Other studies had also shown the low job satisfaction of RHWs in China (1, 2, 31, 32). From the existing research, both of them had become an important reason to aggravate the occurrence of turnover behaviors.

In the following section, we further conducted the discussions based on the reference co-citation results and keywords' co-occurrence status. From Figure 5, three high-frequency clusters were summarized and analyzed. “Motivation,” “rural and remote areas,” and “work environment” were three noteworthy topics during the development of the research field.

The “motivation” cluster went from nearly 2005 to 2015. Positive motivation and job-satisfied healthcare staff represent a basis for the success of modern health institutions. One meta-analysis of motivation and retention for developing countries showed that seven major motivational themes could improve healthcare workers' job satisfaction and retention (33). Previous research on whether there were differences in job motivation and job satisfaction between urban and RHWs in Serbia showed that urban healthcare professionals were significantly more motivated and job satisfied than respondents from rural areas and positive motivation would be beneficial to promote job satisfaction (34). Another discrete choice experiment on the retention and motivation of healthcare workers in remote and rural areas of Nigeria also indicated that positive motivation could improve job satisfaction and enhance retention behaviors (35). A number of studies directed at RHWs in China also supported that motivation and job satisfaction were inseparable. Under the condition of insufficient objective resources, strong motivation was an important subjective condition to improve job satisfaction and reduce turnover intention (2, 14, 31, 32, 36–38).

“Rural and remote areas” was another topic worthy of concern. The shortage of healthcare workers combined with low satisfaction is a worldwide problem but is particularly critical in rural and remote areas. One qualitative study in Mali, sub-Saharan Africa emphasized this result prominently, and individual-level factors, unattractive living and working conditions, community recognition and participation, and quality of leadership could all contribute to this phenomenon (39). Although the supply of pharmacists in Australia exceeded the demand, the supply of pharmacists in rural and remote areas was still insufficient, even if their job satisfaction level was high (40, 41). In the remote rural areas of Western China, the job satisfaction of RHWs was significantly lower than that of economically developed provinces or regions in the East (1, 2, 36, 38, 42).

“Work environment” was an emerging topic which sprung up in recent years. A survey for exploring the association between RHWs and work environment in rural Papua New Guinea indicated that work environment and supportive supervision are the most important influences of job satisfaction for rural nurses in a low- and middle-income country; consequently, the provision of a conducive environment requires attention to the aspects of human relationships (43). Another study directed at rural nurses in Brazil had shown that working conditions, infrastructure, forms of access to the workplace, and distance from the decision center were factors that stood out as difficulties of work, and they were also key factors affecting job satisfaction (44). In addition, some studies on Chinese village doctors also found that various indicators of the working environment were important factors affecting job satisfaction (2, 31, 32, 37, 45). However, in the context of the COVID-19 pandemic, there were few studies on the impact of the work environment on job satisfaction, especially in developing countries.

Discovery and exploration of the relationship among influencing factors were carried out in this study. The following three subjects “Job satisfaction,” “Job burnout,” and “Turnover intention” seemed to be the major factors. Job burnout is characterized by three dimensions, namely, emotional exhaustion, depersonalization, and reduced personal accomplishment (46), and is affected by work factors, individual factors, organizational factors, and social factors (11, 47). In the classical turnover theory, turnover intention is usually regarded as an important cognitive process before turnover behavior. It is the most effective antecedent variable to predict turnover behavior, that is, the higher the turnover intention, the greater the probability of an individual taking turnover behavior (20, 48). According to the resource conservation theory, the individual's own resources are relatively limited. When the external environment poses a potential threat to it or the resources are not supplemented accordingly, it will cause the individual to feel pressure and even lead to the occurrence of job burnout. Resignation is the most common behavior for individuals to deal with job burnout and protect their physical and mental resources.

According to our research results, we could clearly get the relationship between the three factors, that was, job satisfaction had a significant negative predictive effect on RHWs' job burnout and turnover intention, and job burnout had a significant positive predictive effect on turnover intention, and we could deduce the relationship among each other in Figure 6C. Several studies were consistent with conclusions in this study. Evidence supported that job burnout of medical workers was closely related to turnover intention, and there was a significant positive correlation between them, namely, the higher the degree of job burnout, the stronger the turnover intention (13, 49–51). One study on the relationship between job burnout and turnover intention of medical workers covering 25 provincial administrative regions from 2007 to 2020 in China showed that the correlation coefficient *R*-value between job burnout and turnover intention reached 0.43, which meant a high correlation effect (49). Job burnout leads to the disappearance of work enthusiasm and alienation from the organization and occupation, which increases the degree of turnover intentions or turnover behaviors (13, 20, 51–53). One survey about primary healthcare providers in rural China using structural equation modeling demonstrated that job satisfaction proved to be negative predictors of turnover intention, whereas reduced personal accomplishment of job burnout was identified as a positive predictor. Another study on job burnout, satisfaction, and turnover intention of primary healthcare staff in Central China also confirmed this conclusion (51).

Several highly cited references of bibliometrics have brought us enlightenment about the status and probable risk factors of global RHWs' job satisfaction. The first-ranked article was published in the *International Journal of Nursing Studies* and has been cited 420 times as of the time of writing (54). It comprehensively reviewed the literature related to nursing turnover and clarified a series of determinants of it, including job satisfaction, burnout, and so on. In particular, it included a number of literatures, such as the analysis of the differences in job satisfaction and turnover rates between urban and rural hospitals and nursing units in the United States (55), the relationships between job satisfaction and turnover intention among primary healthcare nurses in a rural area of South Africa (56), and the predictors of turnover intention of nurses in all rural and remote practice settings in Canada (57). Based on this study, working environment, professional rank, turnover intention, working pressure, and job burnout were all the important influencing factors of RHWs' job satisfaction, and this was consistent with the findings of this article (54–57). The second-ranked study, which was cited 273 times, also explored the variables related to nurses' job satisfaction through meta-analysis and found that job satisfaction was most strongly correlated with job stress, followed by nurse-physician collaboration, and autonomy (58). The third-ranked study, which was published in the journal of *Archives of Internal Medicine* and was cited 210 times, compared career satisfaction across specialties among 12,474 US physicians (59). Unlike most other developing countries whose RHWs had poor job satisfaction status, they had higher job satisfaction in the US and varied across specialty as well as age, income, and region. Similar to the findings of this study, the fourth-ranked study, which was published in *British Medical Journal* (BMJ) and was cited 157 times, found that RHWs in England also had higher job satisfaction and the rise in intentions to quit was due mainly to a reduction in job satisfaction (60). The fifth-ranked study was cited 133 times and found that low job satisfaction, poor opportunities for development, lack of affective professional commitment, or other factors were the reasons why young nurses had often thought of giving up nursing in Finland (61). In short, there is a strong correlation or prediction between turnover intention, job burnout, and job satisfaction among RHWs, and based on the global perspective, the job satisfaction of RHWs in developed countries is generally higher than that in developing countries.

Collectively, the status of RHWs' job satisfaction is a rarely known but increasingly important issue in today's public health affairs. The world is certainly a better place to live than it used to be, with some previously impoverished parts of the world also experiencing positive developments. The RHWs' career status was one of the most important indicators in this topic. By doing this research, the global research status in this field was vividly demonstrated from the scientometric perspective. In addition, job satisfaction and its related affecting or risk factors were identified and deeply analyzed. This study contributed to informing the broader and more specialized audience involved in health policy decisions. We appealed to all regions worldwide to strive to improve the working environment and current situation of rural doctors and make unremitting efforts to achieve health equity in the coming future.

There were also some limitations in this study. First, the database analyzed in our research was limited to the SCI-Expanded of WOS, and we did not include data from other relevant search engines. However, this data source has been recognized for the quality of its papers, which has been widely applied for most scientometric studies. Second, there was a linguistic bias due to the fact that we only included English publications, despite English remaining the most commonly used language in the world. Besides, only article and review were selected as document type for analysis, while these two types may not fully represent all studies in this research domain. However, article and review were considered the mainstream of publications. Referring to the novel strategy of scientometric and several previous articles (62), the future study can conduct the analysis of journal impact to better reflect the correlation between the quantity or quality of publications, and burst analysis to further discover the emerging trends and grasp the research hotspots. Despite these limitations, we were still confident that the findings of this study can provide an effective perspective of rural healthcare workers' job satisfaction from a global insight.

## Conclusion

This study performed a scientometric analysis from 1995 to 2021 in rural healthcare workers' job satisfaction and demonstrated that this research field had great development potential and broad prospects. The amounts of publications increased from 1 in 1995 to 24 in 2021. The most frequent study category was “Health Care Sciences & Services.” The USA, China, Australia, Canada, and the UK are made up of the core research forces. As an emerging topic related to RHWs' job status, job satisfaction and its related affected factors were systematically summarized by cluster and keywords analysis. During the development of this research field, “motivation,” “rural and remote areas,” and “work environment” were three noteworthy topics. We also highlighted the relationship among job satisfaction, job burnout, and turnover intention in this special group, which can provide scholars with potential directions for future topic selection. In addition, the job satisfaction and working environment of RHWs under COVID-19 pandemic should receive more attention in the future.

## Data Availability Statement

The original contributions presented in the study are included in the article/supplementary material, further inquiries can be directed to the corresponding author.

## Author Contributions

YC and YY: conceptualization, material search, data extraction, and writing—original draft preparation. YC, YY, and YiW: methodology. YC, YY, and YuW: data analysis. YC, YY, YuW, and YiW: writing—review and editing. TD: supervision, project administration, and funding acquisition. All authors have read and agreed to the published version of the manuscript.

## Funding

The Medical and Health Science and Technology Innovation Project of Chinese Academy of Medical Sciences: Research on evaluation of medical science and technology innovation and construction of health service system (2016-I2M-3-018). The funding bodies did not play any role in the design of the study and collection, analysis, and interpretation of data and in writing this manuscript.

## Conflict of Interest

The authors declare that the research was conducted in the absence of any commercial or financial relationships that could be construed as a potential conflict of interest.

## Publisher's Note

All claims expressed in this article are solely those of the authors and do not necessarily represent those of their affiliated organizations, or those of the publisher, the editors and the reviewers. Any product that may be evaluated in this article, or claim that may be made by its manufacturer, is not guaranteed or endorsed by the publisher.
